# Genomic Variability among Field Isolates and Laboratory-Adapted Strains of* Leptospira borgpetersenii* Serovar Hardjo

**DOI:** 10.1155/2018/2137036

**Published:** 2018-05-22

**Authors:** Alejandro Llanes, Carlos Mario Restrepo, Pablo Riesgo-Ferreiro, Sreekumari Rajeev

**Affiliations:** ^1^Centro de Biología Celular y Molecular de Enfermedades, Instituto de Investigaciones Científicas y Servicios de Alta Tecnología (INDICASAT AIP), Ciudad del Saber, Panama, Panama; ^2^Guanábana Biodata Analytics Inc., Panama, Panama; ^3^School of Veterinary Medicine, Ross University, Basseterre, Saint Kitts and Nevis

## Abstract

*Leptospira borgpetersenii* serovar Hardjo colonizes cattle kidneys and may occasionally infect humans and other mammals. Strains belonging to two clonal subtypes (types A and B) with marked differences in their pathogenicity in the hamster experimental model have been described for this serovar. Such differences have been attributed to point mutations in individual genes, although those genes have not yet been characterized. In order to better understand genetic variability among* L. borgpetersenii* serovar Hardjo isolates, we sequenced and compared the genomes of two laboratory-adapted strains and three abattoir-derived field isolates of* L. borgpetersenii* serovar Hardjo. Relatively low genetic variability was observed within isolates of the same subtype, with most of the mutations of moderate or high impact found in the laboratory-adapted isolates. In contrast, several differences regarding gene content and genetic variants were observed between the two subtypes. Putative type-specific genes appear to encode proteins associated with functions that are critical for infection. Some of these genes seem to be involved in transcriptional regulation, possibly leading to a distinct regulatory pattern in each type. These results show that changes in regulatory mechanisms, previously suggested to be critical during* Leptospira* speciation, may occur in* L. borgpetersenii*. In addition, the bioinformatics methodology used in this study for variant calling can be useful to other groups working with nonmodel prokaryotic organisms such as* Leptospira* species.

## 1. Introduction

Leptospirosis is a zoonotic disease of global importance caused by the spirochete bacteria from the genus* Leptospira*. Pathogenic* Leptospira* inhabit the renal tubules of a wide variety of mammalian species. The infected host can be a maintenance host that do not manifest clinical disease or an incidental host, which develops mild to severe clinical disease. Based on the antigenic structure of the lipopolysaccharide, these bacteria are conveniently grouped into serogroups and within these serogroups, members are further grouped into serovars [1]. Serovar-specific host adaptation is well known among members of the genus* Leptospira* and the serovar Hardjo belonging to* Leptospira interrogans* and* Leptospira borgpetersenii* species is known to infect cattle kidneys and genital tracts [2].

Early studies classified North American and New Zealand strains of serovar Hardjo as Hardjobovis strains based on identical restriction endonuclease analysis patterns [3, 4]. Later, it was found that two distinct strains, namely, Hardjobovis and Hardjoprajitno, were circulating in cattle in Northern Ireland and Scotland [5]. The virulent forms of the isolates obtained from cow's milk of agalactic cases and aborted fetuses belonged to Hardjoprajitno, whereas the majority of the abattoir isolates were Hardjobovis [6], hence indicating a possible difference in virulence and pathogenicity between these two distinct types. Later, using DNA hybridization techniques, Hardjobovis was placed in* L. borgpetersenii* and Hardjoprajitno in* L. interrogans *species [7, 8].* L. borgpetersenii* serovar Hardjo is seen worldwide in cattle, whereas the ecological niche for* L. interrogans *serovar Hardjo is unknown [9].

Three clonal subtypes (types A, B, and C) of* L. borgpetersenii* serovar Hardjo have been described [10]; however, the subtype C designation is rarely used and it has been later considered to be erroneous [11]. Other studies have attempted to characterize* L. borgpetersenii* strains by various techniques and suggested the existence of heterogenic clonal populations [12, 13]. In a study conducted in the United States in cattle, it was found that 83% of the isolates belonged to serovar Hardjo and the majority (85%) of these isolates were of type A [14, 15]. It has also been shown that both types have differences in pathogenicity in experimental infection in hamsters [16]. Hamsters infected with a subtype A strain established renal colonization and remained asymptomatic with chronic renal infection, while infection with a subtype B strain resulted in a rapidly debilitating disease similar to the acute form typically observed in incidental hosts.

Bulach et al. [17] sequenced and compared the genomes of two* L. borgpetersenii* serovar Hardjo strains, L550 (subtype A) and JB197 (subtype B). The study revealed that these* L. borgpetersenii* genomes are relatively smaller when compared to those of other* Leptospira* species. This genome reduction is associated with an expansion of mobile elements and the loss of several genes involved in signal transduction, exopolysaccharide synthesis, and nitrogen metabolism [18]. It is suggested that this genome erosion in* L. borgpetersenii* has impaired its viability in aquatic environments, resulting in an increased dependence on mammalian hosts for survival. Although several structural differences were observed between both genomes, differences in disease phenotype were attributed to a combination of point mutations in individual genes [17]. Such genes, however, have not yet been characterized.

In recent years, with the advent of next-generation sequencing techniques, several pan-genomic studies have been conducted for the* Leptospira* genus. Fouts et al. [19] sequenced and analyzed the genomes of 20* Leptospira* species from all major taxonomic groups. This study made a large contribution to our understanding on pathogenesis of* Leptospira*, revealing several virulence-associated features exclusively present in pathogenic species such as CRISPR/Cas systems and specific regulatory and secretory mechanisms. In another comprehensive study, Xu et al. [20] sequenced the genomes of 102* Leptospira* isolates from different regions of the world, 10 of them belonging to* L. borgpetersenii*. Relatively high genomic variability was observed among different species, with several events of apparent gene gain and loss during the evolution of pathogenic species from those that are saprophytic. Among the genes that were found to be differentially present in pathogenic and nonpathogenic species there are several members of two-component systems (TCSs), prokaryotic systems involved in regulation of gene expression in response to changes in their environment [21]. TCSs are composed of a histidine kinase capable of sensing particular stimuli and subsequently activate a highly specific intracellular response regulator by phosphorylation. Changes in these and several other genes were suggested to be crucial for adaptation to a variety of environmental and host-related conditions. The loss of several genes associated with carbohydrate and nitrogen metabolism further supports the hypothesis that pathogenic species of* Leptospira*, including* L. borgpetersenii*, are evolving to become increasingly host-dependent and almost parasitic.

In this study, we sequenced and compared the genomes of five* L. borgpetersenii* serovar Hardjo specimens, two laboratory-adapted strains belonging to types A and B, respectively, and three field isolates. The field isolates, belonging to subtype A, were obtained from an abattoir located in Georgia, United States [22]. We characterized and compared the genomic variability within and between the isolates of distinct subtype and biological origin, emphasizing on features that are potentially related to their pathogenicity.

## 2. Materials and Methods

### 2.1. Bacterial Strains, Culture, and DNA Extraction

The laboratory-adapted strains sequenced in this study derived from strains NVSL S 1343 (here labelled LBH-A) and NVSL S 818 (here labelled LBH-B) from the National Veterinary Services Laboratory (Ames, Iowa, United States). The three field isolates (here labelled BK-6, BK-9, and BK-30) were cultured from abattoir-derived cow kidney samples from Georgia, United States [22]. The isolates were grown and maintained in P80-BA liquid oleic media (National Veterinary Services Laboratory, Ames, Iowa, United States). Up to eight passages were performed and cultures from multiple passages were combined for DNA extraction, in order to obtain the optimum concentration of DNA for whole genome sequencing. Genomic DNA from each specimen was isolated using the MasterPure™ Complete DNA and RNA Purification Kit (Epicentre, Wisconsin, United States) following the manufacturer's instructions.

### 2.2. Genome Sequencing, Assembly, and Annotation

Genomic DNA from the five specimens was sequenced at the Georgia Genomics Facility of the University of Georgia by using the Illumina MiSeq technology, following standard protocols. Reads were trimmed to 250 bp by using trimmomatic [23] in order to remove low-quality regions towards the 3′ end. Genome sequences for each specimen were generated by first assembling the reads de novo with SPAdes [24] and then contiguating the de novo contigs into scaffolds with ABACAS [25]. In the contiguation step, the genome of* L. borgpetersenii *serovar Hardjo strain L550 [17] was used as a reference for type A specimens (BK-6, BK-9, BK-30, and LBH-A), while that of* L. borgpetersenii *serovar Hardjo strain JB-197 [17] was used for the type B specimen (LBH-B). Gene models annotated in the corresponding reference genomes were transferred to the newly assembled scaffolds by using RATT [26]. Annotation was manually revised to correct transferred gene models by using Artemis and the Artemis Comparison Tool (ACT) [27].

### 2.3. Variant Calling

Illumina reads from the five specimens were mapped to the corresponding reference genomes by using BWA [28]. Read alignments were preprocessed with Picard v. 2.2.2 (http://broadinstitute.github.io/picard) to remove duplicate reads and resolve format conflicts. Reads were further realigned around indels by using the Genome Analysis Toolkit (GATK) v. 3.5 [29]. Single-nucleotide variants (SNVs) and short indels (under 50 bp) were detected by using a pipeline developed in house. To increase sensitivity in variant detection, we use the consensus of three variant calling algorithms, namely, UnifiedGenotyper and HaplotypeCaller from GATK and the Samtools pileup v. 1.3.1 [30]. The experimental methodology for variant calling followed GATK's best practices, adapted to work with bacterial genomes in general and with* Leptospira* genomes in particular. Considering that such genomes are haploid, we set the ploidy to 1 and used the multiallelic and rare variant calling model as opposed to the consensus model, which assumes biallelic sites. To deal with the high abundance of transposons in* Leptospira* genomes, reads mapping in multiple positions were not excluded from the variant calling analysis. The per-Base Alignment Quality (BAQ) algorithm was used to reduce the rate of false positive SNV calls [31]. Due to the lack of a curated set of known variants for the species, variant filtering was performed by using arbitrary cut-off values for the technical parameters relevant for each variant calling algorithm, set by evaluating their distribution on the aggregated variants (see Results). After filtering, variants from the three algorithms were merged and annotated. Only variants predicted by the three algorithms and passing the filtering step were selected for annotation. Functional annotation was done with SnpEff v. 4.2 [32]. CNVnator [33] was further used to detect relatively larger structural variants.

### 2.4. Functional and Comparative Analysis of Protein-Coding Genes

Genes from the newly annotated genomes and those from the reference strains L550 and JB197 were clustered into ortholog groups by using OrthoMCL [34]. Each ortholog group was tested for evidence of selection by comparing models 1 and 2 of the codeml program from the PAML 4 package [35]. Functional domain prediction in protein-coding genes was done with Interproscan [36]. Gene ontology enrichment analysis was performed with Blast2GO [37]. Suspected virulence of gene models was inferred by comparison against the virulence factors database (VFDB) [38], considering only the core component containing experimentally characterized virulence factors. Regulatory proteins were predicted with P2RP [39].

## 3. Results

### 3.1. Genome Sequencing, Annotation, and Variant Calling

Genomic DNA from five* L. borgpetersenii* specimens belonging to the Hardjo serovar was sequenced by using the MiSeq platform from Illumina. De novo assembly with SPAdes resulted in assemblies of ~260 contigs with an N50 size of 45 kb on average. Reference genomes for the corresponding types were then used to contiguate the de novo contigs into pseudochromosomes, with an average size of 3.6 Mb for chromosome I and 0.31 Mb for chromosome II. Chromosome size and GC-content of our assembled genomes are very similar to those of the corresponding reference genomes (Table 1). Genomes assembled as part of this study were submitted to NCBI Genbank under BioProjects PRJNA296689 (LBH-A strain), PRJNA296694 (LBH-B strain), PRJNA296675 (BK-6 isolate), PRJNA296677 (BK-9 isolate), and PRJNA296679 (BK-30 isolate).

Approximately 99.8% of the MiSeq reads from each specimen could be unambiguously mapped to the corresponding reference genomes by using BWA, thus indicating high sample purity. Since the main goal of this work was to compare genomic variability among field isolates and laboratory-adapted strains, we paid special attention to the accuracy of the variant calling step. We used the consensus of three independent algorithms, with an ad hoc variant filtering configuration for each of them. As a starting point for filtering, we used the cut-off values for critical parameters recommended by Jia et al. [40] and then refined those values for each algorithm by evaluating their distribution aggregated across all samples (Figures S1 and S2). This approach allowed us to optimize cut-off values for technical parameters relevant to variant filtering, including the variant calling quality and the depth of coverage per variant position (Table S1). Counts of filtered variants per specimen classified according to their predicted biological impact are shown in Table S2. A principal component analysis (PCA) of SNV data clearly separated the specimens into three categories, corresponding to the subtype A field isolates and the laboratory strains from subtypes A and B, respectively, with 80% of the variability among these categories explained by the first two components (Figure S3).

### 3.2. Sequence Variants among Members of the Same Type

**Figure 1:**
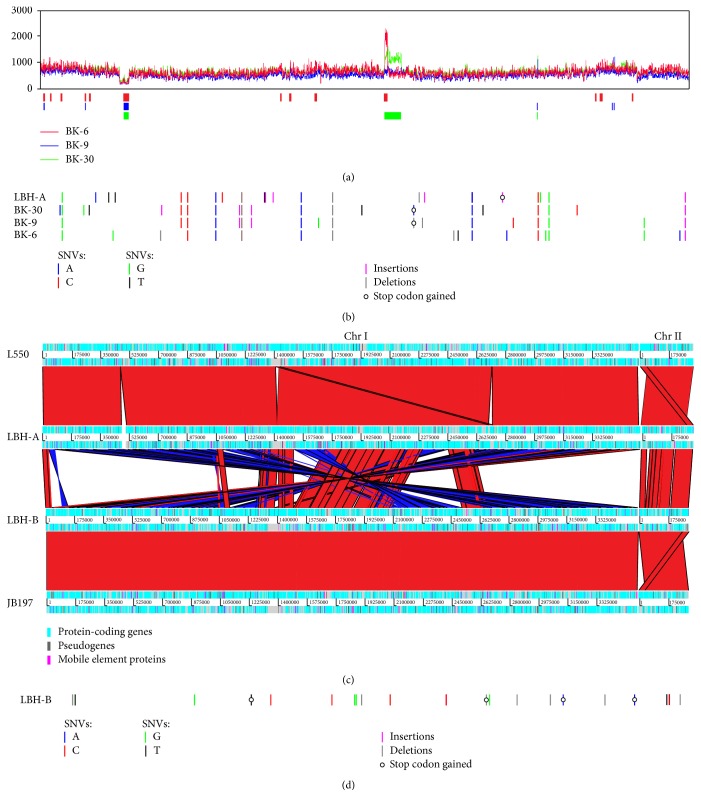
*Overview of genomic variability among the specimens included in this study*. Panel (a) shows variations in read depth for the alignment of reads from the three field isolates against the chromosomes of* L. borgpetersenii* strain L550 (subtype A). CNVs (insertions and deletions) larger than 2 kb predicted by CNVnator are shown below the plot. Panel (b) shows nonsynonymous SNVs and short indels located within protein-coding genes, identified for the four subtype A specimens by using strain L550 as a reference. Panel (c) shows chromosome-to-chromosome comparisons among the two reference genomes for subtype A and B and the genomes of our corresponding laboratory strains. In these comparisons, red bands indicate similar regions and blue bands indicate inversions. Panel (d) is similar to panel (b) but for variants identified for the subtype B laboratory strain, using the genome of strain JB197 as reference.

**Figure 2:**
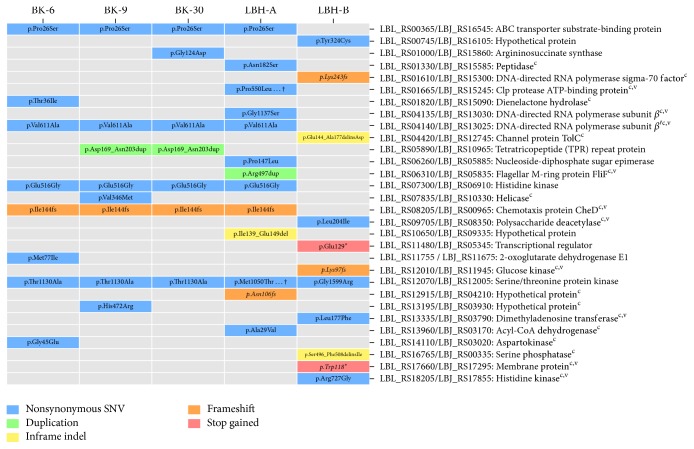
*Reference genes with at least one variant of moderate to high predicted impact in our sequenced specimens of the corresponding subtypes*. Core genes are indicated by a (c) and genes with similarity to previously reported virulence factors are indicated with a (v). Variants that putatively generated a pseudogene in the genome of the corresponding specimen are italic. Variants are labelled by using the protein (p.) part of the HGVS nomenclature (http://www.hgvs.org/mutnomen/). In this notation, “del” means deletion, “ins” means insertion, “dup” means duplication, “fs” means frameshift, and “*∗*” indicates a stop codon gained. † indicates that the corresponding gene has more than one mutation; see Table S3 for the full list of mutations.

We found nearly 60 SNVs per specimen on average, 58–60% of which are located within protein-coding genes. Although we did not observe a notable predominance of either synonymous or nonsynonymous SNVs in any specimen, laboratory-adapted strains seem to have a slightly larger number of nonsynonymous SNVs than field isolates. SNVs and small indels located within protein-coding genes are widespread along the chromosomes and several of them are shared by all specimens of the same subtype or origin (Figures 1(b) and 1(d)). Many of these variants affect genes encoding transposases or other proteins associated with mobile elements, which are expected to have a higher mutation rate due to their unstable nature. In summary, we found 30 protein-coding genes, excluding transposases that are affected by variants with moderate or high predicted impact (Figure 2; Table S3). Twenty of these genes are within the* Leptospira* core genome, as recently defined by Xu et al. [20], and ten of them share sequence similarity with previously reported virulence factors in* Leptospira* and other pathogenic bacteria.

We noticed three identical missense SNVs shared by all of our subtype A specimens, which suggests that such variations may be due to mutations specific to the reference strain L550 rather than to those specimens. In addition to these shared variants, results show relatively few missense mutations exclusive to each specimen. The number of such exclusive mutations is relatively higher in laboratory-adapted strains than in field isolates. In fact, we found only two cases in which a single gene appears to have been affected by more than one missense mutation, both in LBH-A. The corresponding genes, LBL_01665 and LBL_12070, respectively, code for an ATP-dependent Clp protease similar to stress protein ClpC from* Listeria monocytogenes* (VFDB accession code VFG0079) and for a serine/threonine protein kinase. Two additional genes from strain JB197 were found to have orthologs with missense mutations in LBH-B, namely, genes LBJ_RS08350 and LBJ_RS17855. Notably, LBJ_RS17855 codes for a histidine kinase similar to the LetS kinase from* Legionella pneumophila* (VFDB accession code VFG1888), a member of a TCS involved in the induction of the transmission phenotype when nutrients become scarce, especially under laboratory conditions [41].

Variants related to mutations with a higher predicted impact, such as nonsense or frameshift mutations, were also mostly observed in the laboratory-adapted strains. One of such variants, a frameshift mutation affecting a gene encoding a chemotaxis protein of the CheD family, is also apparently specific to strain L550 and causes the corresponding gene (LBL_RS08205) to be slightly shorter than those from our four type A specimens. In addition to this shared variant, we found a frameshift mutation in LBH-A which disrupted and possibly inactivated the ortholog of gene LBL_RS195, whose function is unknown. Conversely, in LBH-B, there are three frameshift mutations in the orthologs of genes LBJ_RS00335, LBJ_RS15300, and LBJ_RS11945 from strain JB197, which encode a methyl-accepting serine phosphatase, a DNA polymerase sigma-70 factor and a glucose kinase, respectively. Mutations associated with the last two genes appear to have originated two corresponding pseudogenes in LBH-B. However, the one affecting the serine phosphatase is not likely to have inactivated the corresponding LBH-B ortholog, since it does not affect segments encoding the HAMP and PPM-like phosphatase domains typical of such proteins [42].

Excluding those affecting transposase genes, we only found nonsense mutations prematurely introducing stop codons in two genes from LBH-B, whose orthologs in strain JB197, respectively, code for a transcriptional regulator (LBJ_RS05345) and an integral membrane protein of the TerC family involved in heavy metal ion resistance (LBJ_RS17295). The later gene, LBJ_RS17295, belongs to the* Leptospira* core genome as defined by Xu et al. [20] and its putative ortholog in* E. coli* is considered a virulence factor [43].

Some of the genes mentioned before have been previously found to exhibit positive selection in studies considering orthologs from several* Leptospira* species [20, 44]. However, we did not find evidence of positive selection in any of these genes when considering only* L. borgpetersenii* orthologs from the corresponding subtypes (data not shown).

### 3.3. Sequence Variants among Members of Different Types

To comparatively describe the differences between type A and type B genomes, we first looked for genes that are putatively specific to each type, whose orthologs were apparently lost or converted into pseudogenes in all the genomes of the opposite type (Table S4). We found 44 genes exclusively present in the type B genomes, half of them coding for transposases or proteins related to mobile elements. Of those genes not associated with mobile elements, 8 are within the* Leptospira* core genome. Three of them, in turn, have sequence similarity to previously reported virulence factors in bacteria. Gene LBJ_RS11430 is similar to the FbpC subunit of an iron (III)-specific ABC transporter from* Neisseria meningitidis* (VFDB accession code VFG1206), while genes LBJ_RS00510 and LBJ_RS13855 are similar to the histidine kinase component of two previously described TCSs. Gene LBJ_RS00510 is similar to a flagellar sensor histidine kinase FleS from* Legionella pneumophila* (VFDB accession code VFG043346) and gene LBJ_RS13855 is similar to kinase YjcC from* Salmonella enterica* (VFDB accession code VFG0584).

In addition to these suspected virulence factors, a gene putatively encoding a glycerate kinase in the subtype B genome (LBJ_RS09170) is apparently inactivated in subtype A, thus suggesting that glycerol metabolism may be affected in this type. Other subtype B genes that have become pseudogenes in subtype A specimens and are associated with metabolic pathways are those encoding a haloacid dehalogenase (LBJ_RS17075) and a methylmalonyl-CoA mutase (LBJ_RS11290), although these genes appear to have putative paralogs with similar function in the genome.

We found 48 genes exclusively present in the genomes of all the subtype A specimens. These include only 8 genes putatively encoding transposases and the number of these genes is notably lower when compared to those found in the genomes of subtype B specimens. As in subtype A, 7 of the genes not associated with mobile elements are within the* Leptospira* core genome and two of them, genes LBL_RS13565 and LBL_RS11610, were found to share sequence similarity with previously reported virulence factors. Gene LBL_RS11610 encodes protein similar to PilH from* Pseudomonas aeruginosa* (VFDB accession code VFG1226). PilH acts as the response regulator of a TCS involved in regulation of flagella-independent twitching motility. Conversely, LBL_RS13565 putatively codes for a single-stranded DNA-binding protein (SSB) reported as a virulence factor in* Salmonella enterica* (VFDB accession code VFG000576).

Inactivation of the ortholog of gene LBL_RS13565 in strain JB197 has been recently reported by Martins-Pinheiro et al. [45] in a study conducted to characterize DNA repair genes in* Leptospira*. SSB is involved in recombinational repair of double-strand break damage in DNA and is thought to be associated with natural competence. Naturally competent bacteria appear to express two copies of the gene encoding SSB, while noncompetent bacteria express only one [46]. Presence of the corresponding two copies of this gene has been observed in most species of* Leptospira*, including the saprophytic nonpathogenic* L. biflexa* [18]. Consequently, loss of one of the two copies in strains JB197 and LBH-B suggests that the natural competence of subtype B strains may be affected.

Other subtype A genes that have been apparently lost in subtype B and may have an implication in virulence and survival are those encoding three additional TCSs members (LBL_RS02170, LBL_RS03580, and LBL_RS10150), two transcriptional regulators (LBL_RS04960 and LBL_RS15495), a cation transporter (LBL_RS09835), an adhesin (LBL_RS14550), an endoflagella protein (LBL_RS13610), and two hydrolases (LBL_RS01270 and LBL_RS01810).

In addition to those genes that appear to be exclusively present in subtypes A and B, we also evaluated the differences between both subtypes at the level of point mutations and small indels. In order to find variants potentially specific to subtype A specimens, we used the same methodology described before to align the reads from subtype A specimens against the subtype B reference genome (strain JB197) and to subsequently call for variants from read alignments. As expected, this resulted in a larger number of variants, including both SNVs and indels (Table S5). Of all the 3,553 genes annotated in the genome of strain JB197, 788 (22%) appear to have an ortholog in at least one of the subtype A specimens with a minimum of one mutation of impact predicted to be high or moderate. When considering only missense mutations altering the chemical nature of the underlying residue, the number of genes with variants was reduced to 483 (Table S6), excluding previously discussed pseudogenes. We found no evidence of positive selection in genes exhibiting variants when considering only* L. borgpetersenii* orthologs.

**Figure 3:**
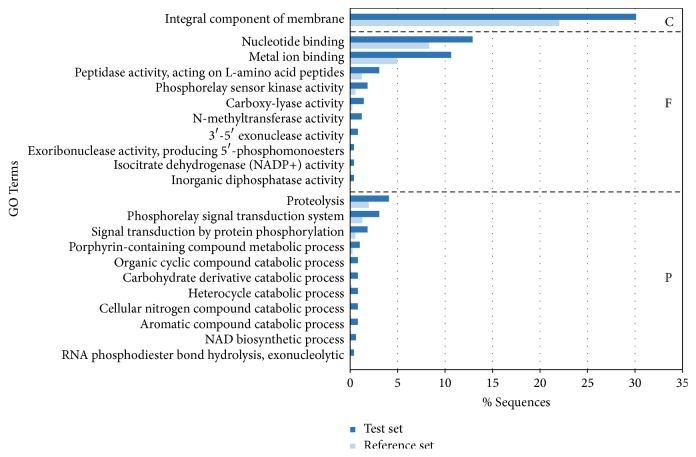
*GO enrichment analysis for the subtype A genes accumulating high-impact mutations*. The analysis was conducted with the genes from the type A specimens having at least one mutation of predicted high impact (test set) and their corresponding orthologs from strain JB197 (reference set). Plot shows GO terms found to be overrepresented with a *p* value below 0.05, ordered by percent sequences in descending order and grouped by GO class (C: cellular components, F: molecular functions, and P: biological processes).

We also looked for gene ontology (GO) terms enriched in the selected genes when compared to all the genes in the reference genome. The refined enrichment analysis resulted in 22 enriched terms, 10 representing molecular functions, 11 representing biological processes, and one representing a cellular component (Figure 3; Table S7). The only cellular component found to be enriched was that associated with integral membrane proteins. This was also the term encompassing the largest number of proteins in the set, including 147 genes encoding a wide variety of membrane transporters, chemotaxis proteins, and peptidases. Several terms in the categories of molecular functions and biological processes, such as phosphorelay sensor kinase activity, phosphorelay signal transduction systems, and signal transduction by protein phosphorylation, can also be associated with TCSs and other regulatory proteins.

Due to the relatively high variability observed in genes encoding proteins involved in transcriptional regulation, such as members of TCSs, we used the P2RP server to assess the number of genes in the reference genomes of both types putatively encoding transcriptional regulators. Results showed that for TCSs, on average, there are at least 36 loci encoding the histidine kinase component and 26 loci encoding response regulators per genome (Table S8). Of the 62 genes predicted for the subtype A reference genome, 14 are within those found to have variants of predicted high impact and selected for the enrichment analysis described before, most of them encoding sensor histidine kinases. The server predicted nearly 80 loci encoding additional transcription factors per genome (Table S9), including sigma factors of RNA polymerase and other types of proteins with DNA-binding domains. Likewise, 10 of these genes are within those previously found to have at least one high-impact variant.

### 3.4. Structural Rearrangements and Copy Number Variations (CNVs)

In addition to SNVs and small indels, we found relatively few structural variants between subtypes. Genomic regions that could be assembled de novo for our five specimens show high synteny with the corresponding reference genomes, thus maintaining the pattern of large-scale rearrangements previously described for the two reference genomes [17]. Two notable exceptions are the transposition of a segment of 24 kb in chromosome I of LBH-A and another one of ~40 kb from chromosome II of both LBH-A and LBH-B (Figure 1(c)). However, gene content and synteny within these segments are not globally altered by their putative rearrangement.

We additionally looked for duplications or deletions in the genomes of subtype A field isolates by using CNVnator. The program predicted relatively few variations larger than 2 kb (Table S10), many of which appear to be flanked by or at least associated with mobile elements. The only deletion we found in the three isolates correspond to one of the two copies of the cluster encoding ribosomal protein genes, which was previously reported to be duplicated in strain L550 [17]. This finding suggests that our field isolates, and LBH-A, do not have that duplication. Since assembly was performed by using the genome of strain L550 as a reference for subtype A specimens, the region for the first copy in strain L550 was replaced by an assembly gap by ABACAS (Figure 1(c)).

Two notable amplifications were found beginning in approximately the same position in the genomes of isolates BK-6 and BK-30, but with different estimated length and copy number. The presence of such variations was confirmed by the observation of a sudden increase in read depth shown in Figure 1(a). In isolate BK-6, the amplified region spans 15 kb and includes 14 protein-coding genes. According to the estimated read depth, this amplification possibly exists in two copies in addition to the region encoded in the chromosome. A much larger region was found to be amplified in isolate BK-30, beginning in approximately the same position of that reported for BK-6, but spanning around 97 kb and encompassing 84 protein-coding genes. Read depth estimates suggest that this amplification exists in only one additional copy. No amplifications of comparable length and read depth were found in the genomes of the laboratory-adapted strains included in this study.

## 4. Discussion

Cattle are known to be the maintenance host of* Leptospira borgpetersenii* serovar Hardjo worldwide. Previous studies have found large differences when comparing the genome of* L. borgpetersenii* with those of other pathogenic* Leptospira* species [17, 20]. Two subtypes of* Leptospira borgpetersenii* serovar Hardjo have been described for this serovar, namely subtypes A and B, with marked differences in disease phenotype in the hamster experimental model. Bulach et al. [17] suggested that phenotypic differences between both subtypes are due to a particular combination of point mutations in several genes, but such genes have not yet been characterized. In addition, little is known about the genetic variability within strains of* Leptospira borgpetersenii* belonging to the Hardjo serovar.

In this study, the genomes of three field isolates and two laboratory-adapted strains of* Leptospira borgpetersenii* serovar Hardjo were sequenced and analyzed. Our goal was to study the genetic variation among field isolates in comparison to laboratory-adapted strains, to improve our understanding of the evolutionary dynamics of this pathogenic species. The field isolates used in this study have undergone multiple passages, but at least one of them (BK-6) has been previously shown to induce colonization in hamsters [22]. Multiple passages may increase the chances of mutations in these isolates, but are very unlikely to affect gene content.

To ensure accuracy in exploring genetic variation, we used a pipeline combining three variant calling algorithms, with customized cut-off values for relevant technical parameters used to filter the detected variants before merging the results. This allowed us to increase sensitivity and to discard potentially false positive calls. In terms of SNVs and small indels, we found relatively few variants associated with mutations of predicted moderate or high impact within the subtype A field isolates and between laboratory strains and their corresponding reference genomes.

The number of variants associated with protein-coding genes (excluding those related to mobile elements) was slightly higher in the two laboratory strains than in field isolates. In the laboratory strains, some of these variants are related to high-impact mutations, including frameshifts and nonsense mutations putatively inactivating the corresponding genes. This higher number of variants in laboratory-adapted strains can be attributed to the lack of selective pressure when growing in laboratory conditions, compared to those strains surviving in the environment and hosts, which need to overcome potentially unfavorable environmental conditions and destructive host defense mechanisms. This distinct evolutionary process developed in laboratory and nature has also been reported for* L. interrogans* [47].

Nevertheless, the number of variants found among the subtype A field isolates and laboratory strains in this study are lower than those we previously found between a field isolate and a laboratory-adapted strain of* L. interrogans* serovar Hardjo [44]. Although it seems that there is relatively low genetic variation in terms of SNVs or small indels within specimens of the same subtype or origin, differences observed in this study allowed separation of specimens into well-defined clusters in a principal component analysis. Unfortunately, when describing genetic variation among field isolates, we were limited to subtype A specimens, since we did not obtain any subtype B isolates from our prevalence studies.

It is worth mentioning that we found a region in chromosome I of subtype A field isolates that appears to be amplified with varying length and estimated copy number. Regardless of the specimen, amplified regions appear to be flanked by mobile elements, suggesting their role in amplification. We have no evidence for the nature of such suspected amplifications, either intrachromosomal or episomic (extrachromosomal), but excision of a region of a chromosome in the form of a plasmid-like amplicon has been previously reported in* Leptospira* [48]. We found no gene putatively related to plasmid replication within the reported amplified regions, but the regions contain several genes that are involved in survival and colonization of host tissues, thus suggesting that their amplification may be associated with a higher dosage for those genes and may represent an adaptive advantage.

To better understand the differences in the genetic background of subtypes A and B, we conducted a careful revision of potentially type-specific genes, complementary to that performed by Bulach et al. [17]. Recent progress in microbial molecular biology, especially on the field of bacterial transcriptional regulation, allowed us to assign a function to some of those genes that were apparently lost in each subtype. In fact, some of those genes appear to encode proteins involved in the regulation of gene expression, including canonical transcriptional regulators or members of two-component systems (TCSs). TCSs are prokaryotic systems composed of a histidine kinase able to sense external stimuli, subsequently activating a highly specific response regulator by phosphorylation [21].

For a better characterization of the genetic differences between subtypes, we mapped the reads from all of the subtype A specimens and used the same protocol described above for variant calling. As expected, this resulted in a reasonably larger number of variants, with counts relatively similar for all specimens and variant types. An enrichment analysis conducted with subtype B genes whose orthologs in subtype A have at least one moderate- to high-impact mutation showed enrichment of GO terms also associated with TCSs, including signal transduction by protein phosphorylation and phosphorelay signal transduction systems. The term phosphorelay refers to the ability many hybrid histidine kinases have to autophosphorylate in an internal receiver domain, before transferring the phosphate group to their corresponding response regulator [49]. In addition to these terms associated with transcriptional regulation, other terms that were found to be enriched are those related to proteolytic activity and membrane processes. Such membrane processes include membrane transport, motility and chemotaxis, all of which are critical for bacterial colonization, invasion and survival within the animal host.

Globally, our findings support that the differences in virulence between subtypes A and B are due to several mutations in individual genes, some of which encode regulatory proteins, possibly leading to a distinct regulatory pattern in each subtype. TCSs play a key role in allowing bacteria to sense, respond and adapt to external stimuli, while they are growing in the environment or colonizing a host [21]. It has been recently shown that loss of certain TCSs and gain of new ones have occurred during the evolution of the* Leptospira* genus [20]. It is believed that such variation in TCSs have been a critical step in the evolution of* Leptospira* species from the saprophytic and nonpathogenic phenotype to those with the ability to colonize animal hosts. We also found that variations in these and other genes involved in transcriptional regulation also appear to occur within strains of the same species and serovar in* L. borgpetersenii*. These findings suggest that future research regarding pathogenomics of* L. borgpetersenii* and perhaps other pathogenic* Leptospira* species should be oriented towards unravelling the mechanisms for regulation of gene expression, some of which have been just recently described in other microbial organisms.

## 5. Conclusions

Our results show relatively low variability at the sequence level within specimens of the same subtype in* L. borgpetersenii* serovar Hardjo, with the majority of the differences observed in laboratory-adapted strains. Several differences were found between subtypes, including putative specific genes and several high- or moderate-impact mutations affecting orthologs. Many of the genes exhibiting differences between subtypes are associated with survival and virulence, including genes encoding proteins involved in transcriptional regulation of gene expression such as canonical regulators or TCSs. These results suggest that changes in regulatory mechanisms also occur in* L. borgpetersenii* at the levels of strains. Genome-wide analysis data from this study will promote our understanding of the evolutionary dynamics and host adaptation factors of this important zoonotic pathogen. In addition, the bioinformatics methodology used in this study for variant calling can be useful to other groups working with nonmodel prokaryotic organisms such as* Leptospira* species.

## Figures and Tables

**Table 1:** Basic features of the five genomes sequenced in this study, compared to those of the reference genomes for *L. borgpetersenii* serovar Hardjo subtypes A and B.

Feature	Reference genomes		Specimens from this study				
			Type A				Type B
	L550 (type A)	JB197 (type B)	BK-6	BK-9	BK-30	LBH-A	LBH-B
Total size (Mbp)	3.93	3.88	3.97	3.95	3.95	3.93	3.88
Chr. I	3.61	3.58	3.65	3.63	3.63	3.61	3.58
Chr. II	0.32	0.30	0.32	0.32	0.32	0.32	0.30
GC-content (%)	40.23	40.24	40.22	40.21	40.21	40.21	40.23
Protein-coding genes	3,623	3,553	3,546	3,531	3,519	3,524	3,522
In mobile elements	154	172	101	100	100	112	140
Transfer RNA genes	37	37	37	37	37	37	37
Ribosomal RNA	5	5	3	3	3	3	3
Pseudogenes	223	232	202	200	191	197	224

## Data Availability

Genomes assembled and annotated as part of this study have been submitted to NCBI GenBank under BioProjects PRJNA296689 (LBH-A strain), PRJNA296694 (LBH-B strain), PRJNA296675 (BK-6 isolate), PRJNA296677 (BK-9 isolate), and PRJNA296679 (BK-30 isolate). The bioinformatic pipeline used in this study is publicly available on GitHub (https://github.com/priesgo/leptospira-variant-calling/releases/tag/v1.0).

## Supplementary Materials

Supplementary 1 Figure S1: distribution of values of relevant technical parameters for predicted SNV aggregated across all samples. Optimized cut-off values used for variant filtering are shown with a blue dashed line. Predicted variants within the regions shaded in pink were discarded after filtering (PDF 131 KB).

Supplementary 2 Figure S2: distribution of values of relevant technical parameters for predicted indels aggregated across all samples. Optimized cut-off values used for variant filtering are shown with a blue dashed line. Predicted variants within the regions shaded in pink were discarded after filtering (PDF 79 KB).

Supplementary 3 Figure S3: principal component analysis (PCA) conducted with moderate- and high-impact variants predicted within samples of each type. The analysis clearly separated field isolates and laboratory strains of the same type (a), with ~80% of the variability from each class explained by the first two principal components (b) (PDF 49 KB).

Supplementary 4 Table S1: cut-off values used for technical parameters in variant filtering (XLSX 10 KB).

Supplementary 5 Table S2: summary of small variants detected between the sequenced specimens and their reference genomes (XLSX 11 KB).

Supplementary 6 Table S3: reference genes with at least one variant of moderate to high impact in the specimens sequenced in this study (XLSX 14 KB).

Supplementary 7 Table S4: putative subtype-specific genes (XLSX 15 KB).

Supplementary 8 Table S5: summary of small variants detected for subtype A genes when using the subtype B genome as reference (XLSX 10 KB).

Supplementary 9 Table S6: genes from* L. borgpetersenii* strain JB197 whose putative orthologs in subtype A specimens have at least one mutation of moderate to high impact (XLSX 38 KB).

Supplementary 10 Table S7: Gene Ontology (GO) terms found to be enriched in the genes from strain JB197 whose orthologs in our subtype A specimens have high-impact mutations (XLSX 15 KB).

Supplementary 11 Table S8: P2PR predictions of members of two-component systems (TCS) in the genomes of* L. borgpetersenii* strains L550 and JB197 (XLSX 15 KB).

Supplementary 12 Table S9: P2PR predictions of transcriptional regulators in the genomes of* L. borgpetersenii* strains L550 and JB197 (XLSX 15 KB).

Supplementary 13 Table S10: structural variants (duplications/deletions) larger than 2 kb predicted by CNVnator in the genomes of the three subtype A field isolates (XLSX 12 KB).
