# Supplementary material for: Genomic Variability among Field Isolates and Laboratory-Adapted Strains of Leptospira borgpetersenii Serovar Hardjo
Source: Int J Microbiol. 2018 May 22;2018:2137036. doi: 10.1155/2018/2137036 (PMC5987247; doi:10.1155/2018/2137036)
Supplement: Supplementary 1 — Figure S1: distribution of values of relevant technical parameters for predicted SNV aggregated across all samples. Optimized cut-off values used for variant filtering are shown with a blue dashed line. Predicted variants within the regions shaded in pink were discarded after filtering (PDF 131 KB). [file 2137036.f1.pdf]

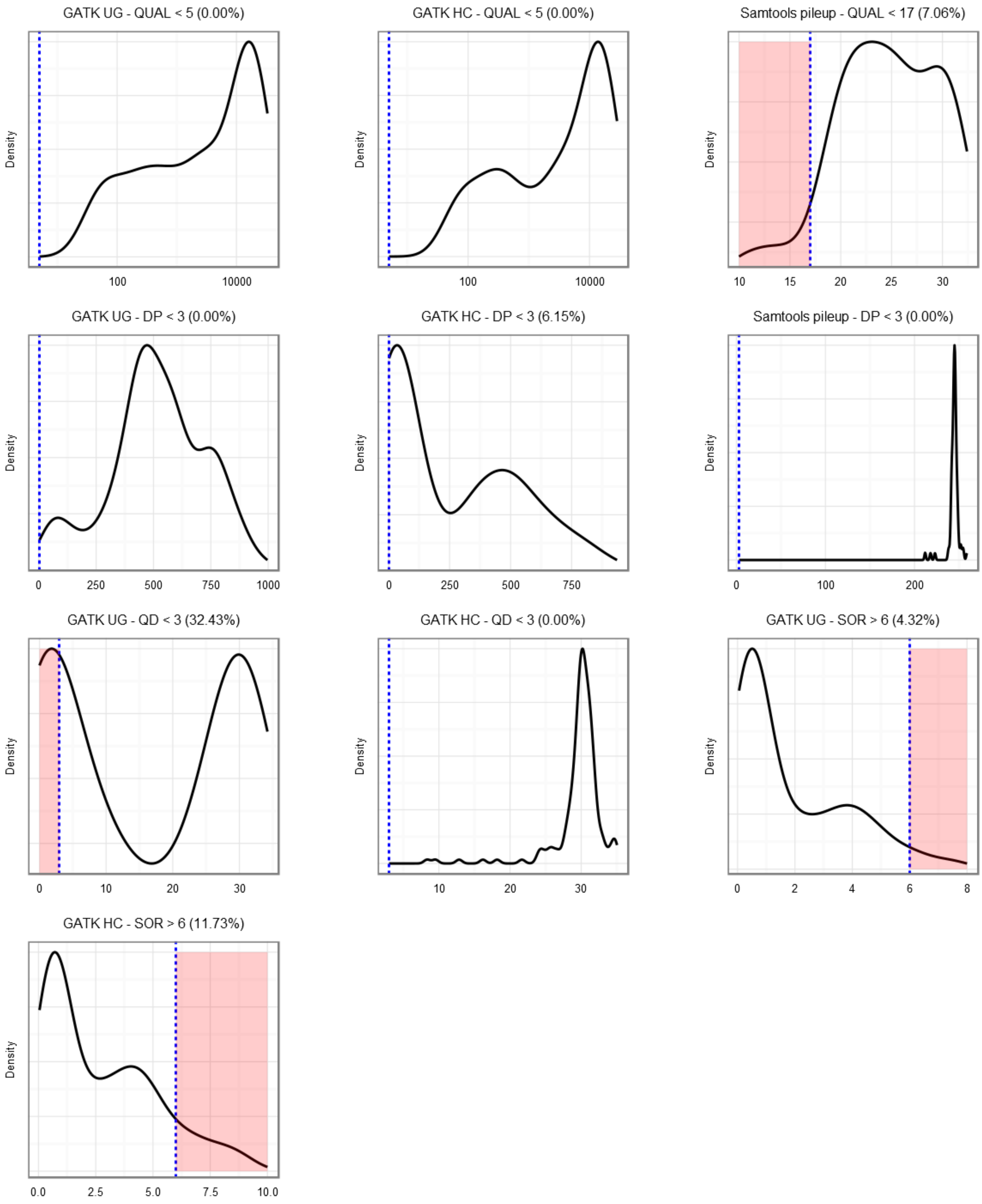

Fig. S1. Distribution of values of relevant technical parameters for predicted SNV aggregated across all samples. Optimized cut-off values used for variant filtering are shown with a blue dashed line. Predicted variants within the regions shaded in pink were discarded after filtering.
