# Supplementary material for: Genomic Variability among Field Isolates and Laboratory-Adapted Strains of Leptospira borgpetersenii Serovar Hardjo
Source: Int J Microbiol. 2018 May 22;2018:2137036. doi: 10.1155/2018/2137036 (PMC5987247; doi:10.1155/2018/2137036)
Supplement: Supplementary 3 — Figure S3: principal component analysis (PCA) conducted with moderate- and high-impact variants predicted within samples of each type. The analysis clearly separated field isolates and laboratory strains of the same type (a), with ~80% of the variability from each class explained by the first two principal components (b) (PDF 49 KB). [file 2137036.f3.pdf]

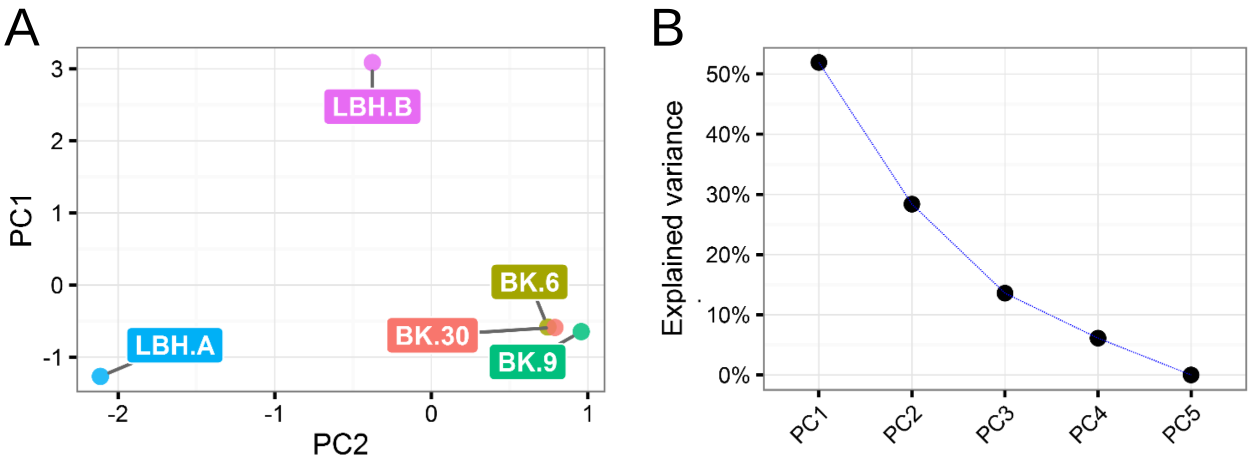

Fig. S3. Principal component analysis (PCA) conducted with moderate- and high-impact variants predicted within samples of each type. The analysis clearly separated field isolates and laboratory strains of the same type **(A)**, with ~80% of the variability from each class explained by the first two principal components **(B)**.
